# Urban and rural differences in geographical accessibility to inpatient palliative and end-of-life (PEoLC) facilities and place of death: a national population-based study in England, UK

**DOI:** 10.1186/s12942-019-0172-1

**Published:** 2019-05-06

**Authors:** Emeka Chukwusa, Julia Verne, Giovanna Polato, Ros Taylor, Irene J Higginson, Wei Gao

**Affiliations:** 1 Department of Palliative Care, Policy and Rehabilitation, Cicely Saunders Institute, King’s College London, Bessemer Road, Denmark Hill, London, SE5 9PJ UK; 2 Knowledge and Intelligence (South West), National End of Life Care Intelligence Network, Public Health England, Grosvenor House, 2 Rivergate, Temple Quay, Bristol, BS1 6EH UK; 3 Monitoring Analytics (Mental Health, Learning Disability and Substance Misuse), Care Quality Commission (CQC), 151 Buckingham Palace Road, London, SWIW 9SZ UK; 4 Royal Marsden NHS Hospital Trust, London, SW3 6JJ UK; 5 Hospice UK, 34-44 Britannia Street, London, WC1X 9JG UK

**Keywords:** Place of death, Rural–urban, Geographic accessibility, Inpatient palliative and end of life care (PEoLC) facilities

## Abstract

**Background:**

Little is known about the role of geographic access to inpatient palliative and end of life care (PEoLC) facilities in place of death and how geographic access varies by settlement (urban and rural). This study aims to fill this evidence gap.

**Methods:**

Individual-level death data in 2014 (N = 430,467, aged 25 +) were extracted from the Office for National Statistics (ONS) death registry and linked to the ONS postcode directory file to derive settlement of the deceased. Drive times from patients’ place of residence to nearest inpatient PEoLC facilities were used as a proxy estimate of geographic access. A modified Poisson regression was used to examine the association between geographic access to PEoLC facilities and place of death, adjusting for patients’ socio-demographic and clinical characteristics. Two models were developed to evaluate the association between geographic access to inpatient PEoLC facilities and place of death. Model 1 compared access to hospice, for hospice deaths versus home deaths, and Model 2 compared access to hospitals, for hospital deaths versus home deaths. The magnitude of association was measured using adjusted prevalence ratios (APRs).

**Results:**

We found an inverse association between drive time to hospice and hospice deaths (Model 1), with a dose–response relationship. Patients who lived more than 10 min away from inpatient PEoLC facilities in rural areas (Model 1: APR range 0.49–0.80; Model 2: APR range 0.79–0.98) and urban areas (Model 1: APR range 0.50–0.83; Model 2: APR range 0.98–0.99) were less likely to die there, compared to those who lived closer (i.e. ≤ 10 min drive time). The effects were larger in rural areas compared to urban areas.

**Conclusion:**

Geographic access to inpatient PEoLC facilities is associated with where people die, with a stronger association seen for patients who lived in rural areas. The findings highlight the need for the formulation of end of life care policies/strategies that consider differences in settlements types. Findings should feed into local end of life policies and strategies of both developed and developing countries to improve equity in health care delivery for those approaching the end of life.

**Electronic supplementary material:**

The online version of this article (10.1186/s12942-019-0172-1) contains supplementary material, which is available to authorized users.

## Background

Place of death has been an important proxy outcome measure of end of life care [1, 2] and can potentially determine the quality of care patients receive prior to death. Access to palliative care is important [3, 4] and a priority of the World Health Organisation, who made a global resolution in 2014, calling on member states to improve access to palliative care as a core component of health [5]. In order to achieve this goal, an understanding of the role of geographic access to inpatient PEoLC facilities in place of death is essential for service improvement, development and planning.

A growing body of international and UK studies have found considerable rural–urban variations in place of death. In general, researchers have found that there is limited use of PEoLC services in rural areas prior to death [6–8] and rural dwellers are more likely to die in hospices or at home compared to urban dwellers [9–13]. These variations suggest that rural and urban dwellers experience different patterns of geographic access to PEoLC facilities.

Access is a complex and multidimensional concept [14–16] comprising of five dimensions; availability, accessibility, affordability, acceptability, and accommodation [14, 17]. Access can also be classified into potential and realised access. Realised access is the actual utilisation of health services, whereas potential access quantifies the propensity to utilise health services [18]. Geographic access hugely influences health service utilisation and outcomes [19–22], but, little is known about the role of geographic access to PEoLC in place of death.

Previous PEoLC accessibility studies in the UK and elsewhere have focused largely on mapping equity of PEoLC provision [23, 24] quantifying proximity to PEoLC [25], identifying underserved or shortage areas of PEoLC facilities [26, 27], characterising of variations in access [23, 28] and location planning or site suitability assessment [29–31]. None of these studies have examined the role of geographic access to PEoLC in place of death. Further, most PEoLC accessibility studies [23, 24, 28, 31, 32] used aggregate-level data as the unit of analysing geographic access by measuring distance or travel time from centroids of census areas, blocks or dissemination area to the nearest PEoLC facilities. The use of aggregate-level data for accessibility analysis can be prone to errors [33, 34] and can potentially lead to inappropriate policies. The study by Gatrell and colleagues [35], is an exception. The authors examined the role of geographic access to PEoLC facilities in place of death, using cancer only data from a specific health authority in North West England, UK and they did not control for important factors (e.g. number of contributory causes of death and region of residence) known to influence place of death in previous studies. Further, the study did not consider whether access varies between rural and urban areas, thus limiting the generalisability of their findings.

Our study addressed these research gaps by using large individual-level death data (including deaths from cancers and non-cancers) to explore the urban–rural differences in the association between geographic access to inpatient PEoLC facilities in England, UK. Understanding the role of geographic access in place of death is the first step to providing quality palliative care. Knowledge of the role of geographic access and how geographic access differs between urban and rural areas can provide an evidence-base for developing robust service improvement interventions to improve access to PEoLC.

## Methods

### Study design and setting

A national population-based observational study in England, UK.

### Data sources and study population

Death data consisting of all deaths from non-accidental causes of patients aged 25 years and above in England, 2014, were extracted from the Office for National Statistics (ONS) death registry. The ONS death registration data included the age and gender of the deceased, the postcode of place of death (i.e. hospital, own home, care home, hospice etc.), the postcode of usual place of residence, the underlying Cause of Death (CoD), and the Number of Contributory causes of Death (NCoD). Death data were linked to the ONS postcode directory file [36] to derive the settlement of the deceased. Settlement classification in the ONS postcode directory file was based on the 2011 Rural-Urban Classification (RUC2011) [37]. The RUC2011 defines an urban area as a settlement with a population of 10,000 people or more [37]. The linkage of death data was based on patients’ postcode of residence.

**Fig. 1:**
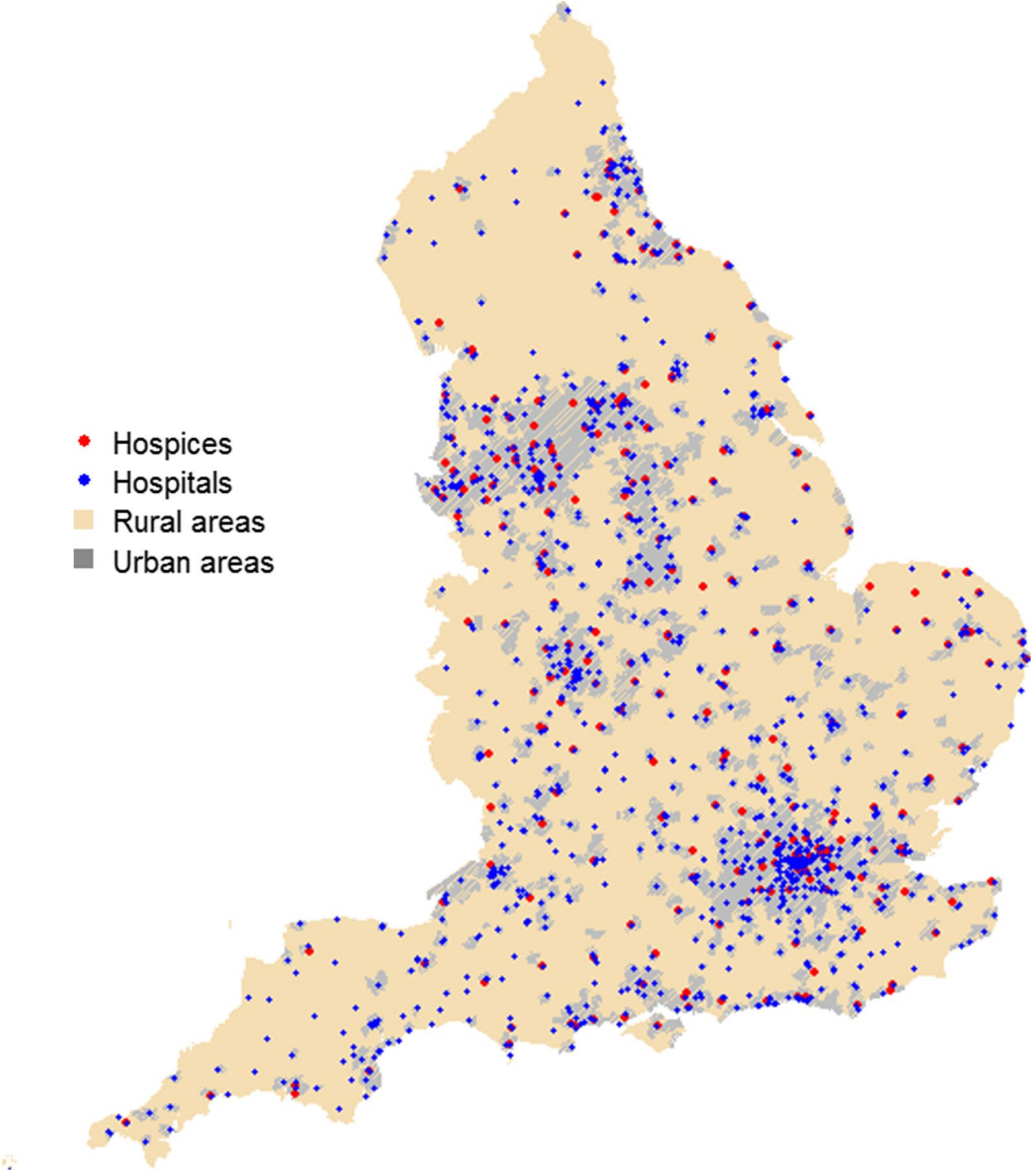
Geographic distribution of inpatient PEoLC Facilities in England, UK. The digital boundary file contains National Statistics data© Crown copyright and database right (2016) and contains Ordnance Survey data© Crown copyright and database (2016)

Hospital location data were downloaded from NHS digital (https://digital.nhs.uk/) [38], and the National End of Life Care Intelligence Network website (http://www.endoflifecare-intelligence.org.uk/home) [39]. Hospice location data were provided by Hospice UK and supplemented with data from the UK Hospice aid directory website (http://www.hospiceaid.org.uk/images/guide_to_hospice_aid/hospicedirectorybycounty.pdf) [40]. Hospice data used in this study comprised of hospices providing adult inpatient services. Children’s hospices were excluded from the datasets. Figure 1 shows the spatial distribution of hospices and hospitals in England, UK. Road data used for measuring geographic access was the Ordnance MasterMap^®^ integrated transport network layer [41].  All datasets were checked and cleaned for errors and the final datasets comprised of 184 adult inpatient hospices and 1226 hospitals.

### Outcome, explanatory and confounding variables

Place of death was the outcome variable categorised into five groups; hospital, hospice, home, care home and other places. In our model, we focussed on the top three commonplace of deaths: hospices, hospital, and home [42].

Potential geographic access was the explanatory variable, categorised into four groups (0–10 min, 10–30 min, 30–50 min and over 50 min). Access was analysed as a categorical variable to facilitate interpretation and comparison of results between models. Potential geographic access was derived by measuring drive times from patients’ place of usual residence to the nearest inpatient PEoLC facility. We accounted for differences in speed limits of various road types (e.g. A-roads, B-roads, Minor roads and Motorways). Calculation of drive time was completed in ArcGIS 10.5 using the Network Analyst extension [43]. The process involved estimating travel times between sets of origin locations (i.e. patients’ residential locations) and destinations (Inpatient PEoLC facilities) along a road network. Results are stored in an origin–destination matrix, consisting of drive time values from each patient’s place of residence to hospitals (1226 by 430,467) and hospices (184 by 430,467). Minimum drive time values calculated from O-D matrices were linked to patients’ records as proxy estimates of patients’ access. Estimation of drive times from patients’ residential address offered a detailed analysis of patients’ potential geographic access to PEoLC facilities, compared to area-based access measures used in other PEoLC accessibility studies.

Confounding variables comprised of age at death (25–54, 55–64, 65–74, 75–84 and 85 +), gender (male and female), marital status (married, single, divorced, widowed and not given or others), CoD, NCoDs, Index for Multiple Deprivation (IMD), and  region of residence. IMD was used as a proxy for patients’ socioeconomic status. It is an area-based measure of deprivation, with domains in income, employment, education skills, training, health, disability, housing/services barriers and living environment. The IMD score for each patient was derived by linking patients’ residential postcode to their Lower Super Output Areas (LSOAs) of residence, this was calculated for all LSOAs. LSOAs are census units with an average population of 1500 people [44]. The IMD was based on data for 2010 and was grouped into quintiles of 1 (most deprived) to 5 (least deprived). CoD and NCoDs were based on the 10th revision of the International Classification of Diseases (ICD-10) coding systems (Table 1).

**Table 1:** Patients’ socio-demographic and clinical characteristics of adults who died in rural and urban areas, England 2014 (N = 430,467)

Variable	Value	Rural	Urban
N (%)		86,432 (20.1%)	344,035 (79.9%)
Geographic access (min)	Median drive time to hospice (minimum, maximum)	17.9 (0.16, 93.9)	9.02 (0.01, 105.4)
	Median drive time to hospital (minimum, maximum)	9.71 (0.02, 73.9)	4.5 (0.00, 76.2)
Age	25–54	3.7	5.4
	55–64	6.7	8.1
	65–74	16.6	16.7
	75–84	29.9	30
	85 +	43.2	39.7
Gender	Female	51.3	52.3
	Male	48.7	47.7
Marital status	Divorced	7.8	10.3
	Married	43.1	37.7
	Separated/dissolved	0.1	0.1
	Single	7.1	9.6
	Unknown/not stated	0.3	0.5
	Widowed	41.6	41.8
Cause of death (ICD-10 codes)	Cancers (C00–C97)	30.1	30.4
	*CBDs (G45–G46, I60–I69)	7.6	7.1
	*COPDs (J40–J44, J47)	5.7	5.9
	* CVDs (I00–I52, I70–I99)	21	21.1
	Neurological conditions (G35–G37, G20, F02.3, G12)	1.7	1.7
	Others (not in the above categories)	33.8	33.7
Number of contributory Causes of deaths (NCoDs)	0	25.2	23.4
	1	28.1	26.9
	2	21.4	22
	3	12.9	14.1
	4	7	7.6
	5 +	5.4	6.1
Index of Multiple (IMD)	1 (most deprived)	3.3	24.1
	2	12.9	21.6
	3	29.2	18.5
	4	30.7	17.6
	5 (least deprived)	24	18.3
Regions	East	17.6	9.9
	East-midlands	12.5	8.1
	London	0.3	12.6
	North-east	5.3	5.8
	North-west	8.3	16
	South-east	18.6	16
	South-west	18.7	9.5
	West midlands	9.5	11.3
	Yorkshire and The Humber	9.4	11
Place of death	Other place	1.2	1.1
	Hospice	5.5	6.2
	Home	24.6	22
	Care home	25.5	22.3
	Hospital	43.1	48.4

*Cause of death: Cardiovascular Diseases (CVDs), Chronic Obstructive Pulmonary Disease (COPD) and Cerebrovascular Disease (CBDs)

### Statistical analysis

Data on patients’ socio-demographic and clinical characteristics were described using percentages. Maps were used to visualise geographic access in urban and rural areas. Maps were derived from the aggregates of individual-level median drive time to inpatient PEoLC facilities in each Clinical Commission Group (CCG). CCGs are planning regions for commissioning health services in England, UK [45]. There were 211 CCGs in England in 2014.

A modified Poisson regression model with robust error variances [46] was applied to evaluate the association between geographic access and place of death. Two models were developed for patients in rural and urban areas. Model 1 compared the association between geographic access to hospice, for hospice death (1) versus home death (0). Model 2 compared the association between geographic access to hospitals, for hospital death (1) versus home death (0). Only deaths falling within comparison groups were included in the models.

Models were adjusted for patients’ socio-demographic and clinical characteristics (Table 1). The magnitude of the association between geographic access and place of death were described using adjusted prevalence ratios (APRs) and 95% Confidence Intervals (CIs). Model goodness of fit was based on the difference between model-based estimate and actual data. All models showed a statistically significant reduction in deviance values (*p* < 0.001), suggesting an adequate fit to the data. All statistical analyses were completed in R. version 3.3.1 (www.r-project.org).

## Results

### Patients’ socio-demographic characteristics

After removing invalid drive time values, with zero drive time from hospice and hospitals (approximately 0.3%), the final dataset consisted of 430,467 adult deaths. More than three-quarters of the deaths occurred in urban areas (79.9%) and about one-fifth (20.1%) in rural areas. Hospital was the most common place of death in rural (43.1%) and urban areas (48.4%). Proportions of death increased with increasing age in rural and urban areas, with patients aged 85 and over accounting for the largest proportion of death in rural (43.2%) and urban areas (39.7%). Cancer accounted for 30.1% of deaths in rural and 30.4% in urban areas. Cardiovascular Diseases (CVDs) accounted for an almost equal proportion of deaths in urban (21.1%) and rural areas (21.0%). Deaths from Chronic Obstructive Pulmonary Disorders (COPDs) were marginally higher in urban areas (5.9%), compared to rural areas (5.7%). Deaths varied remarkably across regions. The South West (18.7%) and the South East (18.6%) were the regions with most rural deaths. London (0.3%) and the North East (5.8%) had the lowest proportion of deaths in rural areas.

### Geographic access to inpatient PEoLC facilities in urban and rural areas

**Fig. 2:**
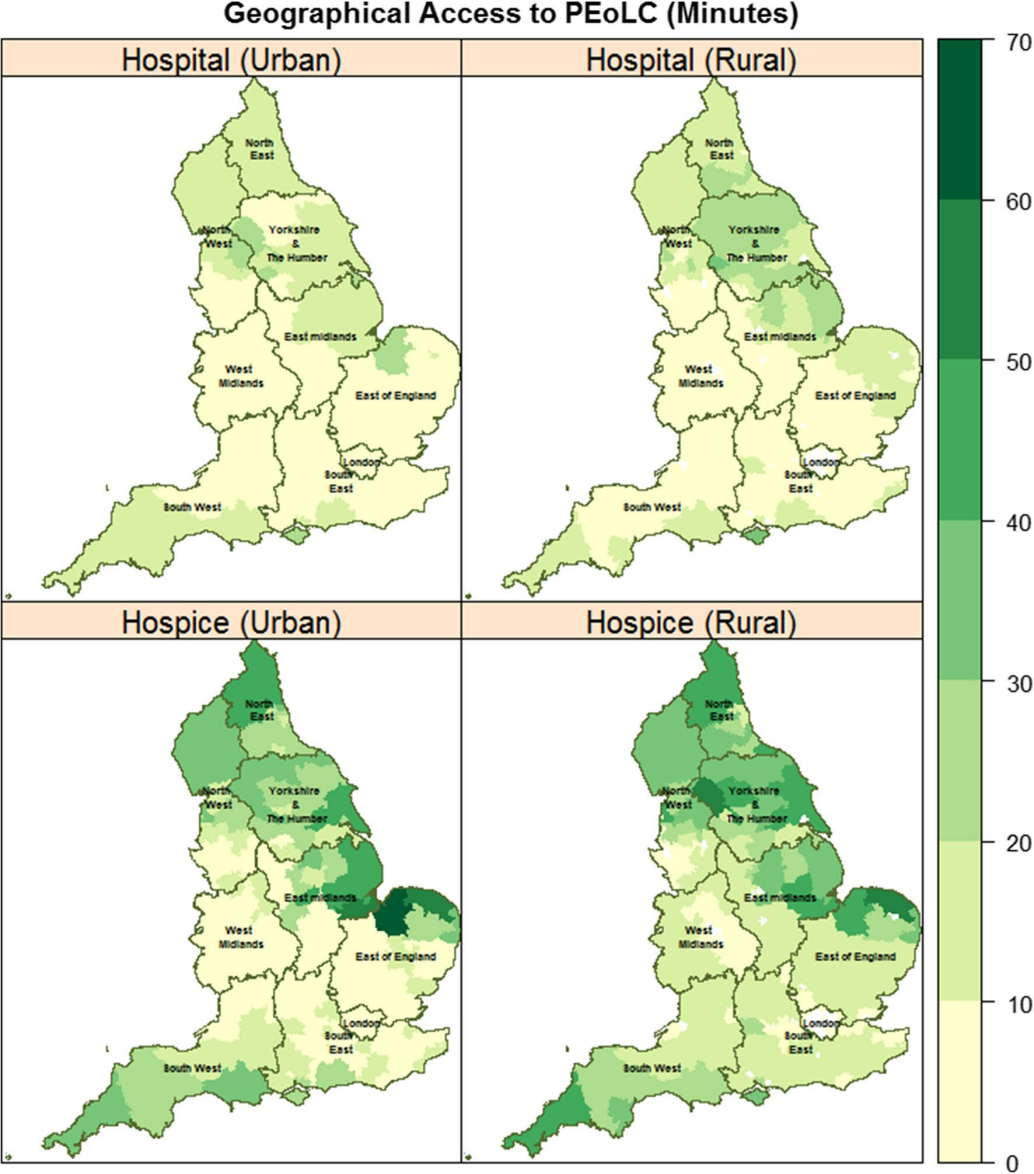
Geographic access to inpatient PEoLC Facilities in England, UK. Maps shows the aggregate CCG level median nearest drive time (min) to PEoLC-related facilities within regional boundaries. Access to hospitals in urban areas (top-left), access to hospitals in rural areas (top-right), access to hospice in urban areas (bottom-left) and access to hospice in rural areas (bottom-right). The digital boundary file contains National Statistics data© Crown copyright and database right (2016) and contains Ordnance Survey data© Crown copyright and database (2016)

There are distinct rural–urban differences in geographic access to inpatient PEoLC facilities across CCGs in England (Fig. 2). Overall, patients in the urban area had better geographic access, compared to rural areas (Table 1). In terms of access by inpatient PEoLC facility (Table 1), patients who died in the hospital had relatively easier access to hospital in both rural (median drive time: 9.7 min) and urban areas (median drive time: 4.5 min), compared to patients who died in hospices in rural (median drive time: 17.9 min) and urban areas (median drive time: 9 min).

### Relationship between geographic access to inpatient PEoLC facilities and Place of Death in rural and urban areas

Results of multivariable analysis (Table 2), show an inverse relationship between place of death and drive time in both rural and urban areas (results of complete models are given in Additional file 1: Tables S1 and Additional file 2: S2). In Model 1 (Hospice vs Home) there was a strong inverse association, with a dose–response effect. In urban areas, patients who lived within a drive time range of 10–30 min from hospices were less likely to die in a hospice (APR: 0.83, 95% CI 0.81–0.86, *p* < 0.001) compared to patients who lived closer (0–10 min). Successive increases in drive time from 10 min to 30–50 min (APR. 0.74, 95% CI 0.70–0.77, *p *< 0.001) or over 50 min (APR. 0.50, 95% CI 0.43–0.59, *p* < 0.001), resulted in corresponding reductions in likelihoods of hospice death. The effects were greater in rural areas for all drive time categories (APR: 0.80, 95% CI 0.76–0.85, *p* < 0.001[10–30 min]; APR. 0.64, 95%. CI 0.58–0.70, *p* < 0.001 [30–50 min] and APR. 0.49, 95% C I 0.43–0.56, *p *< 0.001 [Over 50 min]).

**Table 2:** Association between geographic access and place of death in rural and urban areas

Models	Geographic access (min)	Rural		Urban	
		Unadjusted PRs (95% CI)	Adjusted PR (95% CI)	Unadjusted PRs (95% CI)	Adjusted PR (95% CI)
1	Drive time to hospices				
Hospice versus home	0–10 min	Ref	Ref	Ref	Ref
	10–30 min	0.77 (0.72–0.81)***	0.80 (0.76–0.85)***	0.84 (0.82–0.86)***	0.83 (0.81–0.86)***
	30–50 min	0.57 (0.52–0.62)***	0.64 (0.58–0.70)***	0.73 (0.70–0.77)***	0.74 (0.70–0.77)***
	Over 50 min	0.42 (0.36–0.48)***	0.49 (0.43–0.56)***	0.47 (0.40–0.54)***	0.50 (0.43–0.59)***
2	Drive time to hospitals				
Hospital versus home	0–10 min	Ref	Ref	Ref	Ref
	10–30 min	0.99 (0.97–1.00)*	0.98 (0.96–0.99)***	0.98 (0.97–0.99)***	0.99 (0.99–1.00).
	30–50 min	0.99 (0.96–1.02)	0.95 (0.92–0.98)**	0.96 (0.93–0.98)**	0.97 (0.95–1.00)*
	Over 50 min	0.80 (0.58–1.12)	0.79 (0.59–1.06)	0.94 (0.82–1.09)	0.98 (0.86–1.13)

Prevalence ratios (PRs) were estimated from modified poisson regression. PR > 1 indicates a higher likelihood of death at hospice or hospital compared to the reference category. PR < 1 suggest lower likelihood of hospice or hospital death compared to the reference category (Ref - reference group). Adjusted PRs were derived by adjusting for age, cause of death (COD), gender, marital status, Index of Multiple Deprivation (IMD), number of contributory cause of deaths (NCODs) and regions. Triple asterisks (***) denotes *p* value less than 0.001, double asterisks (**) is *p* value less than 0.01 and Single asterisk (*) is less than 0.05 and a dot (.) means *p* value less than 0.1

The effect of drive time on hospital deaths (Model 2: Hospital vs Home), was similar to those obtained for hospice in rural and urban areas. In urban areas, patients who lived more than 10 min drive time from a hospital location (10–30 min APRs. 0.99, 95% CI 0.99–1.00, *p* = 0.057; 30–50 min, APRs. 0.97, 95% CI 0.95–1.00, *p* = 0.032; over 50 min, APR. 0.98 95% CI 0.86–1.13, *p *= 0.79), were less likely to die in a hospital, compared to patients who lived closer (i.e. 0–10 min). The magnitudes of the APRs (APRs. 0.98, 0.96–0.99, *p* < 0.001 [10–30 min]; APRs. 0.95; 95% CI 0.92–0.98, *p* < 0.01 [30–50 min]; APRs. 0 .79, 0.59–1.06, *p* = 0.11 [Over 50 min]) were lesser in rural areas.

## Discussion

The results of this population-based study show that geographic access to inpatient PEoLC-facilities is associated with place of death, with the magnitude of the effect being greater in rural areas compared to urban areas. These findings warrant the formulation of end-of-life care policies that account for differences in settlement types, such that outreach home care may become especially important in more rural areas.

Patients in rural and urban areas who lived more than 10 min away from hospices were less likely to die in a hospice, compared to those who lived within 10 min, drive time. Even after adjusting for patients’ socio-demographic and clinical characteristics (Model 1), the associations remained statistically significant (*p* < 0.001). Similar results were found in the association between geographic access to hospital and place of death. However, the gradient was not as steep as those obtained in geographic access to hospice. Our results are consistent with the findings of Gatrell and colleagues [35]. Their study showed that the likelihood of dying in a hospice or hospital was inversely associated with distance, using cancer only data in the North West England and, controlled for a limited number of confounders. By contrast, our study used large national death data (consisting of deaths from Cancers and Non-cancers) and adjusted for patients’ socio-demographic and clinical characteristics including, CoD, NCoDs and regions of residence. Although, the inclusion of covariates did not change the direction of the association, but the strength of the association was reduced slightly in the adjusted model (Table 2). This implies that ignoring these important covariates may exaggerate the effect of geographic access to PEoLC in place of death.

Our results show a clear dose–response relationship between geographic access to hospice and hospice deaths in urban and rural areas (Model 1). This suggests that greater drive time from a hospice leads to a corresponding decline in the likelihood of dying in that hospice. This attenuating effect of drive time on the likelihood of hospice death, suggests that the further away patients lived from a hospice, the lower the chance of hospice death. The same is true for geographic access to hospitals (Model 2). However, we would exercise caution against the over interpretation that this means that patients who lived in rural areas and died at home had less good care, as it is possible that areas with limited geographic access to inpatient facilities are served by community-based care, especially in rural areas.

The differences in the magnitude of the effect between urban and rural areas underscores the importance of place of settlement on where people die. One possible reason for variations may be due to differences in the spatial organisation of inpatient PEoLC facilities in rural and urban areas. As Fig. 1 shows, hospitals and hospices are clustered in urban areas, compared to rural areas. This means that in rural areas, patients experience longer drive time to hospices and hospitals. In addition, the map of aggregate CCG level, median drive time to inpatient PEoLC facilities (Fig. 2) shows that patients in rural areas travel longer distances to use hospice and hospital services, compared to their urban counterparts. Northern Norfolk around King’s Lynn was a notable exception (Fig. 2, bottom-left). Drive time to hospice in the area appears to be greater in urban areas (approximately above 60 min from hospice locations) compared to rural areas. This is perhaps due to limited number of hospices in relation to the proportion of urban deaths (Fig. 1). Further investigation is needed to understand the cause of the drive time anomaly in Northern Norfolk.

Rurality is associated with an increased chance of home death. It will be important to determine whether this is because of choice or because of access problems to inpatient facilities [47], which can be misinterpreted as choice. Several studies have reported similar rural–urban disparities in health services provision/access [7, 28, 48]. Rural–urban disparities in geographic access have implications for services utilisation [7, 8, 49, 50] and can lead to late diseases diagnosis amongst rural dwellers [51, 52].

The findings of this study have important implications for service improvement, planning and delivery, particularly in terms of the way community-based palliative care services are configured to meet needs. Community hospice teams should expand their service catchments farther from hospice and hospital locations, especially in rural areas where drive time to facilities is greater. There is evidence that such a targeted service delivery approach can foster service uptake in health service deficit areas [53]. It could be argued that community services may also need to be increased in urban areas as the higher rate of death in hospitals may not be reflective of choice, but rather a decreased chance of dying at home due to insufficient community services.

Although our study used data from England, UK findings are applicable to other countries. The findings can assist health policy makers in understanding the role of geographic accessibility in place of death and for planning service improvement interventions especially in underserved areas (e.g. rural areas). Findings can feed into local end of life policies and strategies of other countries to improve equity in health care delivery for people nearing end-of-life. For example, formulation of policies to foster decentralisation of inpatient PEoLC services from urban areas can alleviate the challenge of geographic access in rural areas. One way this can be achieved is by introducing non-institutionalised services—e.g. hospice-at-home services. Technology-enhanced care such as telehealth services [54] or video conferencing can improve access to specialist palliative care in remote or rural areas. For example, video conferencing has been piloted in Orkney Scotland Hospice [47] and it is currently being used to address the problem of access in remote communities in Scotland [55]. Increasing services capacity, such as the provision of inpatient hospice beds, and the introduction of initiatives that foster collaborative palliative service delivery across care settings—e.g. delivery of hospice services at care homes or hospitals, should be adopted. There is evidence that such collaborative involvement or partnership can increase out-of-hospital death [56] and improve access to specialist palliative care services [57].

### Strength and limitations of study

To our knowledge, this is the first study to comprehensively examine rural–urban differences in the association between geographic access to inpatient PEoLC facilities and place of death using large individual-level death data. The main strength of this study is the use of individual-level data as the unit of analysis of geographic access. Deriving access from individual-level data is less prone to aggregation errors, compared to aggregate level access measures used in other PEoLC accessibility studies [23, 24, 28, 31, 32], that assumes that individuals within in a geographical area have the same accessibility scores [58]. The ONS death registry provides spatially disaggregate data with patient-level information, such as age, gender, the main cause and contributory causes of death. Linkage of the data with an area-level settlement and socio-economic data enabled the exploration of urban and rural differences and the role of access in place of death. Similar databases can be used in other developed and developing countries. While the quality and completeness of death registers is a fundamental issue in developing countries, a national representative sample of existing death data linked with local census estimates and health facility location data could be used to analyse rural and urban difference in access. The study made important contribution by exploring how access varies between urban and rural settlements, while adjusting for a wide range of covariates that may confound the association between geographic access and place of death not considered in previous study on geographic access and place of death. This is important for more robust service improvement interventions and policy formulation.

Geographic access was derived by measuring the drive time from patients’ place of residence to the nearest inpatient PEoLC facility. Drive time was used because it is a more appropriate measure of travel efforts [59, 60]. It provides a better proxy estimate of geographic access compared to straight-line or ‘crow-fly distance’, although the latter has been shown to be highly correlated with other measures of access [33, 61, 62]. Nevertheless, results should be interpreted with caution, as drive time may give a false impression that patients have access to only one mode of transportation (private car or public transport). In addition, the calculation of drive time was based on fixed speed limits without consideration of road conditions. Studies have shown that speed limits are variable depending on the time of day [63] and seasons [64]. Prospective studies should consider these factors when measuring drive time. Another strength of this study relates to the use of prevalence ratios to estimate relative risk, instead of odd ratios. The latter has been shown to exaggerate effect size when the outcome of interest is common [46].

Our study has some limitations. Due to data processing constraints, we calculated drive time by splitting the datasets based on patients’ region of residence. This involved calculating drive time for each region separately and recombining the datasets. Using this stratified approach leads to edge-effect error [65] as cross-regional movement is not taken into account. For example, patients living in the border of regions may access services outside their region of residence which are closest to them.

Furthermore, geographic access was quantified based on the assumption that patients will visit facilities nearest to them. However, in practice, patients’ usage of service encompasses more than geographic proximity [66]. Access is a multifaceted concept consisting of both geographical and non-geographical factors [17] (e.g. availability, affordability, accommodation, acceptability). Analysing access in the context of geographic dimension ignores non-geographical factors (e.g. the number of beds, workforce and, patients’ preference) that can influence access to inpatient PEoLC facilities. For example, it was not possible to quantify community based generic or specialist palliative care facilities to understand whether they are greater in rural areas. Future studies should incorporate these non-geographical aspects in the model to gain a holistic picture of the role of geographic access in place of death.

## Conclusion

Findings from this study have shown that geographic access is an important determinant of place of death and that the size of the effect differs in rural and urban areas. These results highlight the need for the formulation of end-of-life policies/strategies that consider differences in settlements types. Overall, findings from this study showed that rural dwellers are less likely to die at PEoLC inpatient facilities, compared to their urban counterparts. Findings can feed into local end-of-life policies and strategies of other countries to improve equity in health care delivery for people nearing end of life. The utility of individual-level death data as a unit of analysing geographic access permitted a detailed examination of urban/rural differences in the association between geographic access to inpatient PEoLC and place of death.

## Additional files

**Additional file 1.** Table S1.xls: (Additional file Table 1) Association between Drive time to hospices and Place of death in Rural and Urban areas in England (2014), adjusting for clinical and socio-demographic characteristics of patients.

**Additional file 2.** Table S2.xls: (Additional file Table 2) Association between Drive time to hospitals and Place of death in Rural and Urban areas in England (2014), adjusting for clinical and socio-demographic characteristics of patients.

## Abbreviations

PEoLC: Palliative and end-of-life care facilities
IMD: Index for Multiple Deprivation
CoD: Underlying Cause of Death
NCoD: Number of contributory causes of death
LSOAs: Lower Super Output Areas
ICD-10: International Classification of Diseases 10th revision
CCGs: Clinical Commission Groups (CCGs)
APRs: Adjusted Prevalence Ratios
CI: Confidence Interval
CVDs: Cardiovascular Diseases
COPD: Chronic obstructive pulmonary disease
CBDs: Cerebrovascular diseases
ONS: Office for National Statistics
RUC: Rural–Urban classification
O–D: Origin–Destination matrix
